# Feasibility and acceptability pilot study of an online weight loss program in rural, underserved communities

**DOI:** 10.7717/peerj.18268

**Published:** 2024-10-03

**Authors:** Ashleigh Oliveira, Nouf Alfouzan, Jin Yu, Asma Yahya, Kayla Lammy, Mary Liz Wright, Diane Reinhold, Lisa Peterson, Ashley Brewer, Janet Liechty, Manabu T. Nakamura

**Affiliations:** 1 Department of Nutritional Sciences, University of Illinois at Urbana-Champaign, Urbana, Illinois, United States; 2 Food Science and Human Nutrition, University of Illinois Urbana-Champaign, Urbana, Illinois, United States; 3 University of Illinois, Marshall, Illinois, United States; 4 University of Illinois, Freeport, Illinois, United States; 5 University of Illinois, Hillsboro, Illinois, United States; 6 United States Department of Veteran Affairs, Bloomington, Illinois, United States; 7 School of Social Work, University of Illinois at Urbana-Champaign, Urbana, IL, United States; 8 Carle-Illinois College of Medicine, University of Illinois, Urbana, IL, United States

**Keywords:** Rural, Weight, Obesity, Protein, Fiber, Weight loss, Rural health, Diet, Nutrition, Nutritional geometry

## Abstract

**Background:**

The purpose of this intervention was to investigate the feasibility, acceptability, and preliminary effectiveness of an online weight loss program, EMPOWER, in rural, underserved communities.

**Methods:**

Adults with a body mass index (BMI) ≥ 25 kg/m^2^ living in rural counties were recruited through collaboration with University of Illinois Extension. The intervention lasted 1 year including online educations sessions, nutrition and lifestyle coaching, and diet and weight monitoring *via* a novel web application, MealPlot. Feasibility was measured by enrollment attainment, participant retention, online education session completion, and completion of anthropometric and dietary measures. Acceptability was measured by survey using Likert scales of satisfaction for all program components. Anthropometric measurements, 24-h dietary records, and food frequency questionnaires (FFQs) were measures of program efficacy. Additionally, two interviews were collected for program feedback.

**Results:**

Enrollment of 16 participants was attained, however due to higher than anticipated dropout (retention 62.5%, *N* = 10) at 3-months, 62.5% of the education sessions were completed and 75.0% of anthropometric and dietary measures. The average satisfaction rating for the comprehensive program was 4.2/5 with lowest satisfaction being the MealPlot web application 2.7/5 (*N* = 11). On average a clinically significant (≥5% baseline weight) weight loss of 6.2 ± 6.0% body weight or 5.7 ± 5.3 kg and improvements to protein and fiber intake at 12 months (*N* = 10) were observed.

**Conclusions:**

A novel online weight loss program showed adequate to strong feasibility and acceptability and preliminary results indicating efficacy among a pilot sample of rural residents. Future studies are required to investigate means of improving retention and reducing the burden on program collaborators.

## Introduction

In the year 2020, rural counties of the United States (US) accommodated around 46 million people which was 14% of the population (Dobis et al., 2021). Data from the Behavioral Risk Factor Surveillance Survey (BRFSS) indicates the prevalence of obesity in rural counties surpassed that of metropolitan counties by 5.5% in the year 2016 (CDC, 2018).

Reasons for the development of excess adiposity are highly individual, but there are many socioeconomic and environmental factors prevalent in rural areas that are associated with higher Body Mass Index (BMI) (Trivedi et al., 2015; Woolf & Aron, 2013). For example, income in the US, which is lower in rural areas, is negatively associated with BMI and weight-related comorbidities (Bentley, Ormerod & Ruck, 2018; Cohen et al., 2023). Other resource disparities in rural areas associated with BMI include limited access to healthy and affordable foods, physical activity facilities, educational opportunities, healthcare infrastructure, and barriers to transportation (Cohen, Greaney & Sabik, 2018; Seguin et al., 2014; Lenardson, Hansen & Hartley, 2015). Additionally, rural residents have longer commutes to supermarkets and may be more likely to stock up on bulk food items that can maintain for long periods of time instead of fresh goods (Larson, Story & Nelson, 2009); thus diets in rural areas have been characterized as having a higher fat and calorie content than in urban areas (Befort, Nazir & Perri, 2012).

While research suggests that dietitians are highly effective weight loss program administrators (Verberne et al., 2019, 2020; Bleich et al., 2015; Morgan-Bathke et al., 2022, 2023), access to healthcare in rural areas is limited (Gorczyca et al., 2018; Hewko et al., 2021; Chatterjee, 2022; Pelletier et al., 2020; Pelletier et al., 2020). Access to a dietitian usually requires physician referral at a hospital or a clinic, however, the number of hospitals and physicians working in rural areas is declining (Government Accountability Office, 2017; Nielsen, D’Agostino & Gregory, 2017; Kaufman et al., 2016). Physicians in rural hospitals are more likely to be near retirement and the influx of new physicians to such areas is diminishing, resulting in a net loss (Fraze et al., 2022; Rosenblatt & Hart, 2000). Moreover, many rural hospitals are faced with inadequate funding (Government Accountability Office, 2017). Given that a larger portion of patients in rural areas rely on Medicare and Medicaid, which offer lower reimbursement rates compared to private health insurance, these hospitals face financial challenges and many are incapable of providing preventative services like medical nutrition therapy (Government Accountability Office, 2017; Fraze et al., 2022). As a result, rural residents often do not have access to preventative care nearby. This inconvenience is linked to reduced office visits and poorer health outcomes (Kelly et al., 2016).

Federal entities in the US are actively pursuing large-scale initiatives aimed at mitigating this decline in rural healthcare services. For example, in 2022, the US Department of Agriculture (USDA) allocated $2.7 million in grants to bolster healthcare endeavors in rural Illinois. Despite these endeavors, there remains an absence of comprehensive weight loss programs specifically catered to the needs of rural communities.

Of the few major rural weight loss programs, the MOVE! program, developed for US Department of Veterans Affairs (VA), may be the largest (Goodrich et al., 2018). MOVE! provides a comprehensive weight loss program approach including guidance on diet, physical activity, and behavior change (Goodrich et al., 2018). The VA reports a significant obstacle to success with rural participants is the length of their commute to a MOVE! facility (Maciejewski et al., 2019; Jackson et al., 2015). A proposed solution is telehealth, which serves as one of the available modalities for MOVE!, however its accessibility is confined to certain facilities and is usually preceded by an in-person referral to the program (Robinson et al., 2022). Additionally, the VA reports staffing and training shortages of MOVE! program facilitators resulting in an insufficient number to meet the demand of the population reflective of the healthcare shortage in rural areas (Goodrich et al., 2018).

An innovative dietary weight loss program, Individualized Dietary Improvement Program (iDip) demonstrated promising weight loss results that may be applicable to rural populations (Lee et al., 2022). The program was developed and tested on an in-person platform (Lee et al., 2022, 2024). In a 12-month formative clinical trial of the iDip, the average participant weight loss was clinically significant at −6.5 ± 8.4 % (Lee et al., 2022). The iDip was converted to a fully-online program, EMPOWER that uses nutrition and lifestyle education, nutrition coaching, lifestyle coaching, and a newly-developed web application, MealPlot with the objective of sustainable diet change and rate of weight loss (Morgan et al., 2014). By administering EMPOWER, may address commonly cited barriers to healthcare access in rural areas. Specialty healthcare providers such as dietitians and social workers are available by telehealth, educational materials are available *via* an online platform, and weight and diet monitoring are tracked through visual feedback tools online. Additionally, the program uses an evidence-based Social Cognitive Theory (SCT) approach to foster behavior change (Annesi, 2022; Turner-McGrievy et al., 2020; Morgan et al., 2014). However, little is known about whether the EMPOWER program could be successfully implemented among rural residents.

The primary objectives of the pilot study are to: (1) Evaluate the EMPOWER weight loss program for rural populations through active assessment and mitigation of access barriers, digital literacy assessment, and collaboration with rural partners; (2) assess the feasibility and acceptability of the online weight loss program among rural residents. The hypothesis is that EMPOWER is feasible and acceptable as a means of viability and not statistical significance. Additionally, it is hypothesized the program will show early indicators of efficacy by demonstrating weight loss and improvements in protein and fiber intake.

## Materials and Methods

### Design

This was a single arm quasi-experimental longitudinal study with repeated measures of weight, body composition, and diet intake over 12 months among a convenience sample of rural residents, with 1 year follow-up. Non-randomization was deemed the most appropriate design for this rural pilot study because the primary purpose was to assess feasibility and acceptability of the weight loss program in a rural setting; and resources were allocated for a pilot study. The study was approved by the University of Illinois Institutional Review Board (Ethical Application #22642). The study was registered on ClinicalTrial.gov (#NCT05587790).

### Participants

Participants were recruited from seven Illinois counties with a population of less than 50,000 people. Three Illinois Extension Nutrition and Wellness educators serving these counties assisted with recruitment by means of social media advertising, flyers, and word of mouth. Eligibility criteria included age 18 to 75 years, BMI > 25 kg/m^2^, fluent in English, willingness to weigh daily, and primary residence within one of seven rural counties in Illinois served by three Extension regional offices. Exclusion criteria included being currently pregnant or lactating, diagnosed with severe chronic disease, and previous or planned bariatric surgery. The three Cooperative Extension Service offices were used as locations for collecting anthropometric measurements and as hubs for participant recruitment led by Extension Nutrition and Wellness educators.

### Recruitment

Recruitment flyers were distributed by Extension educators *via* social media websites and physical copies were placed at Extension offices and public bulletin areas. Participants were also invited by word-of-mouth from Extension educators during community networking. Participants that met inclusion criteria were provided with a description of the study and preview of the consent form. Invitation was prioritized to those with higher number of weight-associated comorbidities. For those taking prescriptions for weight-associated conditions, a primary care provider approval was required to participate. Those participants that indicated continued interest scheduled a meeting at their local extension office where baseline measurements and surveys were collected, consent forms signed, and the program instructions were provided by a researcher. Research Electronic Data Capture (REDCap) software was used to collect eligibility criteria, consent, and background information and nutrition surveys, and dietary intake.

### Interventions

#### MealPlot web application

A web application, MealPlot, was developed to increase program accessibility and apply behavior change techniques (BCTs) such as problem solving and self-monitoring to enhance participant outcomes (Aguiar et al., 2022). The application included a page for designing meals, creating 1 day food records (24-h food records), and monitoring weight; and it included several individualized visual feedback tools described below. The application also acted as a portal between researcher and participant allowing collection of weight data and food records for personalized feedback and analysis.

Instead of traditional calorie counting or macronutrient tracking, the primary outcome of the dietary intervention was achieving a calculated protein and fiber density per meal and per day optimized for weight loss without diminished skeletal mass (Lee et al., 2024). By achieving a protein and fiber rich diet, participants may feel a greater sense of satiation which helps achieve a calorie deficit and also the nutrient needs for a safe weight loss can be met (Clark & Slavin, 2013; Koh-Banerjee et al., 2004; Slavin, 2005). Participants’ logged foods were displayed on a Protein Fiber Chart (PF Chart), which is a two-dimensional graph of protein density and fiber density (Fig. S1) (Lee et al., 2022; Pratt et al., 2016). On the PF Chart, the plotted meals are displayed with a target box to direct the participant to the nutritive goals. Thus, the intervention teaches participants how to use an individualized visual decision-making tool, the PF Chart, found in their portal on the MealPlot web application, to achieve the desired PF density ratio that will support successful weight loss. Participants are able to access PF Charts generated from their 1-day food records and they can also use the PF Chart tool interactively for meal planning and decision making.

The MealPlot web application also displayed the participants’ weights on an individualized weight chart (Fig. S2). Participants connected a provided Wi-Fi scale to the MealPlot web application and were asked to weigh daily. The weight chart displayed a graph of the participant’s actual weights, an individualized 3-month goal line, and a static trendline of 1 pound per week loss to reflect the minimum ideal loss rate.

#### EMPOWER eText 2.0

Nutrition and lifestyle education was provided by an online textbook with embedded multimedia and assignments. The platform, eText, was developed by the Center for Innovation in Teaching and Learning at the authors’ institution. The EMPOWER eText 2.0 incorporated adjustments from the previous version such as reorganization of sessions and improved application assignments. Considerations of rural populations unique needs were included in educational sessions on topics such as obtaining, cooking, and storing foods and recipe alternatives to reduce or replace saturated fat and sodium intake. The eText 2.0 product included 17 online educational sessions covering weight loss principles, essential nutrients, physical activity, and lifestyle tips. Each session contained videos with accompanying text and activities requiring up to 45 min per session to complete. Answers to activities were accessible by researchers allowing for feedback and ensuring understanding of the material.

#### EMPOWER rural program intervention

**Figure 1:**
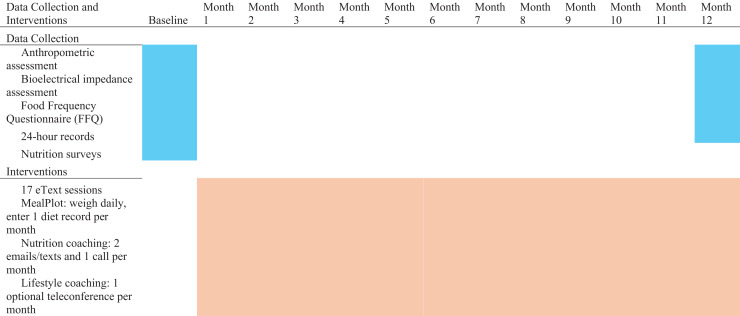
Intervention components and data collection flowchart of the 1-year trial. EMPOWER Rural program consisted of 17 online nutrition and lifestyle education sessions (EMPOWER eText 2.0), daily weighing by Wi-Fi scale, 24 nutrition coaching messages by email or text, 12 nutrition coaching sessions by phone or video call, one lifestyle interview at baseline, and 12 optional lifestyle online support groups and coaching sessions by teleconference. Assessments were collected as baseline and 12 months.

EMPOWER Rural program consisted of 17 online nutrition and lifestyle education sessions (EMPOWER eText 2.0), daily weighing by Wi-Fi scale, 24 nutrition coaching messages by email or text, 12 nutrition coaching sessions by phone or video call, one lifestyle interview at baseline, and 12 optional lifestyle online support groups and coaching sessions by teleconference. The intervention components and data collection flowchart is presented in Fig. 1. The sessions were completed on a flexible schedule adapted to the participant’s availability and understanding of the material. However, minimum participation boundaries to remain in the program were established as completing four eText 2.0 sessions within the first 2 months. Participants weighed themselves daily on a Wi-Fi scale and monitored their progress on the weight chart within the MealPlot app.

Participants individually communicated with a nutrition coach by email or text every other week and by phone or video call at least once per month. The nutrition coach was either a professional registered dietitian or nutrition sciences graduate researcher in the dietetics program. The mode of communication was selected by the participant. Nutrition coaching involved feedback on 24-h records collected through the MealPlot application, eText activity answers, and weighing frequency and weight loss progress. Participants could voluntarily join group lifestyle coaching sessions that provided behavior change coaching using motivational interviewing techniques by teleconference once per month and individual lifestyle sessions as requested. Subject material of the lifestyle coaching was directed by the needs of the participants and was aimed at strategizing ways of adhering to the prescribed diet. Lifestyle coaching and monthly support groups were provided by faculty and graduate student researchers from the School of Social Work at the authors’ institution.

### Outcome measures

#### Internet access and digital health literacy

Internet access and digital health literacy were assessed through a modified survey of the Digital Health Literacy Scale (DHL) and Computer Email Web Fluency Scale (CEW) addressing specific concerns applicable to the EMPOWER Rural program (van der Vaart & Drossaert, 2017; Bunz, 2004). The DHL is a measure of digital literacy skills including how users access the internet and technology and their self-rated skills in various technologies (NIH, 2024). For EMPOWER purposes, questions regarding access and reliability of Wi-Fi were selected from these measures. The CEW assesses self-rated ability to use a computer, email, and navigate the web (Bunz, 2004). Questions were selected from the CEW pertinent to the needs of the study including basic computer, email, and web navigation and excluding higher-level skills unnecessary to the weight loss program. The authors have permission to use these instruments from the copyright holders.

#### Feasibility

Feasibility was a primary outcome operationalized as achieving target enrollment of 16 participants, retention of 80% of participants at 3-months, 75% average completion of eText sessions at 12 months, and 80% completion of study measures including 24-h records, and in-person anthropometric testing at 12 months.

#### Acceptability survey and interview

Acceptability was a primary outcome of all intervention components were evaluated by a 3-months survey questionnaire and interview that was adapted from Batsis et al. (2019) (Article S3). Achievement of at least 4/5 using a 5-point Likert scale was considered acceptable. Acceptability of the lifestyle component was operationalized as 50% or more of the participants using one or more of the optional lifestyle support components (baseline interview, support groups, or coaching).

#### Anthropometrics

Anthropometric measurement protocols were implemented to test capability of data collection in rural settings, as tools for coaching participants, and as an early indicator of efficacy. The changes from baseline were also used as secondary outcomes. Participants met individually with a researcher from the University of Illinois at their nearest Extension education office in a private room to gather height, weight, waist and hip circumferences, and body composition at baseline, 6 and 12 months (Seidell et al., 2001). Height was recorded with shoes removed using a stadiometer (Seca 700, Hanover, MD, US) to the nearest 0.25 inches. Weight and body composition were gathered using a Bioimpedance device (InBody 270 USA, Cerritos, CA, US) to the nearest 0.5 kgs (1.1 lbs). Change in body composition was assessed to monitor feasibility of the study in terms of potential effects of the intervention such as skeletal mass loss, which would require immediate adjustment to the dietary recommendations or withdrawal from the study. Weight was also measured daily by participants themselves using an internet-enabled Wi-Fi scale (Withings, Issy-les-Moulineaux, France) which sent data automatically to the MealPlot application, accessible by both researcher and participant. Waist and hip circumferences were measured to the nearest 0.1 cm using a retractable tape measure (Gulik II; Gay Mills, WI, USA). The waist to hip ratio was calculated based on waist and hip circumference measurements.

#### Diet

Multiple methods of assessing change in dietary habits were implemented. Dietary change was measured by the average change in protein density and fiber density based on an adapted version of the standardized EPIC-Norfolk food frequency questionnaire (FFQ) at baseline and 12 months (University of Cambridge, 2020). Self-reported 1-day dietary records were also collected at baseline and 12 months (NIH, 2024). Alterations in protein, fiber, and calorie intake from FFQ and 24-h records were considered preliminary indicators of potential program efficacy. Diet records made by participants in the MealPlot app were used for nutrition coaching solely as diet guidance.

#### Exit interviews

To further assess participant perspectives on acceptability of the program, a structured exit interview was conducted at 12-months over telephone. The exit interview questions were adapted from Matson et al.’s (2018) survey of program acceptability. Six questions were asked, of which four were close-ended and two open-ended (Article S4). The objective of the closed-ended questions was to pinpoint specific components of the multi-faceted program that needed attention in terms of likeability, efficacy, and accessibility. The objective of the open-ended questions was to probe for general participant perspectives and takeaways.

### Sample size

A sample size of 16 participants was selected based on a recommended sample size for a proof-of-concept pilot study and common practices among feasibility pilot studies in rural communities (Julious, 2005; Batsis et al., 2020; Misra et al., 2021; Coulter, 2018). Additionally, since primary objectives feasibility and acceptability are lacking in powered sample size calculations, the secondary outcome of weight loss (≥5% as indicated by clinical significance) was used to ensure the sample size was justified. A minimum sample of 10 would allow detection of 5% difference with an actual SD of 8.0 (Lee et al., 2022). Considering an average rate of attrition at 22.1%, a sample of 16 was considered sufficient (Borek et al., 2018).

### Statistical methods

Baseline, feasibility, and quantitative acceptability measures were analyzed using descriptive statistics. Feasibility is described as capable of achieving target enrollment of 16 participants, a dropout of less than 20%, and completion of 75% of education sessions and 80% of study measures. Acceptability was considered as a 4/5 by Likert scale on average by program participants. A Shapiro-Wilk test was performed to assess for data normality. Wilcoxon matched pairs sign t-tests were used to determine outcome differences in weight, skeletal and fat mass, and protein and fiber densities at baseline and 12-months. Analyses were conducted for both completers only and the total sample size (using intention-to-treat with missing data input as no change from last collected measure). Data was analyzed using SPSS Statistics version 27 (IBM., Armonk, NY, USA) with a *p*-value of < 0.05 considered statistically significant. Data are reported as mean (SD). Effect size was determined as single-group pretest-posttest design of Cohen’s d for repeated measures (d_RM_) (Lakens, 2013). The transcripts of the 3-months acceptability interview and exit interview were coded and responses were analyzed for indicators of high or low acceptability of each component of the program.

## Results

### Participant flow

**Figure 2:**
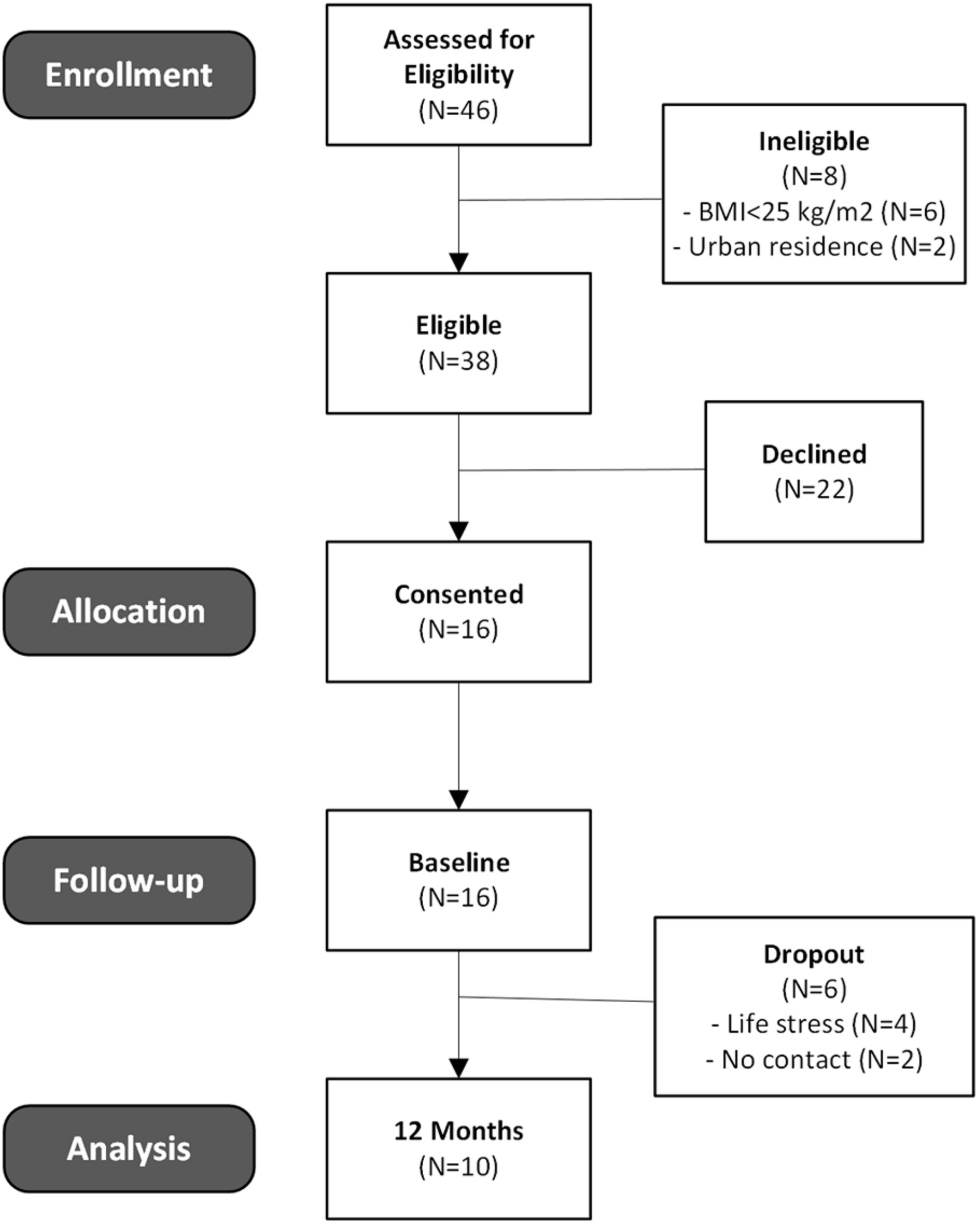
CONSORT diagram of participant eligibility through to final 1-year data collection.

A consort diagram is shown in Fig. 2. Sixteen participants were initially enrolled into the study after 4 weeks of recruitment and completed the baseline surveys and anthropometric data collection. Four participants dropped the program within the first month due to life stressors unrelated to the program. An additional two participants dropped without reported reason by 3 months, and they were unavailable for follow-up resulting in a dropout of 37.5%.

### Baseline demographic data

Table 1 presents baseline participant characteristics of both enrolled and completing participants. The average age of all enrollees was 47 ± 10 years, and the average BMI and weight was 37.5 ± 4.4 kg/m^2^ and 105.2 ± 18.7 kg respectively. Self-reported comorbidities were less than two per person on average with the most common being depression (50.0%) and osteoarthritis (31.3%). All participants were employed either full-time or part-time. Most annual individual income ranged from $50,000–$74,999 (50.0%) or $25,000 –$49,999 (37.5%). Completing participants did not have statistically different baseline characteristics than non-completing participants but did have a lower average baseline weight of 99.9 ± 14.6 kg and two-point lower BMI of 37.5 ± 4.4.

**Table 1:** Baseline participant characteristics. Baseline characteristics of all participants and those participants who completed the entire study. Abbreviations: M, mean; SD, standard deviation.

Characteristics at baseline	All (*N* = 16)	Completers (*N* = 10)
	M ± SD or N (%)	M ± SD or N (%)
Age, years	47 ± 10	47 ± 10
Female, %	15 (93.8%)	9 (90.0%)
Male, %	1 (6.3%)	1 (10.0%)
White, %	16 (100.0%)	10 (100.0%)
Marital status, %		
Single	3 (18.8%)	1 (10.0%)
Married	11 (68.8%)	8 (80.0%)
Divorced	2 (12.5%)	1 (10.0%)
Widowed	0 (0.0%)	0 (0.0%)
Insurance, %		
Medicare	1 (6.3%)	1 (10.0%)
Medicaid	3 (18.8%)	1 (10.0%)
Private insurance	12 (75.0%)	8 (80.0%)
Smoking status, %		
Non-smoker	13 (81.3%)	8 (80.0%)
Former smoker	1 (6.3%)	1 (10.0%)
Current smoker	2 (12.5%)	1 (10.0%)
Education, %		
High school	2 (12.5%)	0 (0.0%)
Some college	6 (37.5%)	4 (40.0%)
College degree	6 (37.5%)	4 (40.0%)
Post-college degree	2 (12.5%)	2 (12.5%)
Employment status, %		
Full-time employment	14 (87.5%)	8 (80.0%)
Part-time employment	2 (12.5%)	2 (20.0%)
Multiple jobs	3 (18.8%)	2 (20.0%)
Variable shift hours	2 (12.5%)	1 (10.0%)
Night shift worker	1 (6.3%)	1 (10.0%)
Individual annual income, %		
Less than $25,000	1 (6.3%)	1 (10.0%)
$25,000–$49,999	6 (37.5%)	3 (30.0%)
$50,000–$74,999	8 (50.0%)	6 (70.0%)
$75,000–$99,000	1 (6.3%)	0 (0.0%)
Anthropometrics		
Weight, kg	105.2 ± 18.7	99.9 ± 14.6
BMI, kg/m^2^	39.2 ± 6.1	37.5 ± 4.4
Waist circumference, cm	117.3 ± 12.4	113.8.2 ± 9.2
Hip circumference, cm	125.9 ± 13.4	120.8 ± 8.4
Waist to hip ratio		
Male (*N* = 1)^c^	–	–
Female (*N* = 16)	0.93 ± 0.04	0.93 ± 0.05
BMI Category^a^		
Class 1 obesity	4 (25.0%)	3 (30.0%)
Class 2 obesity	6 (37.5%)	5 (50.0%)
Class 3 obesity	6 (37.5%)	2 (20.0%)
Comorbidities, %^a^		
Breathing problems	2 (12.5%)	0 (0.0%)
Depression	8 (50.0%)	4 (40.0%)
Diabetes, Type 1	0 (0.0%)	0 (0.0%)
Diabetes, Type 2	0 (0.0%)	0 (0.0%)
Elevated blood pressure	1 (6.3%)	1 (10.0%)
Gastrointestinal disorder	4 (25.0%)	2 (20.0%)
Heart disease	1 (6.3%)	1 (10.0%)
High cholesterol	3 (18.8%)	1 (10.0%)
History of cancer	1 (6.3%)	1 (10.0%)
Hypertension	3 (18.8%)	1 (10.0%)
NAFLD	2 (12.5%)	1 (10.0%)
Osteoarthritis	5 (31.3%)	2 (20.0%)
Prediabetes	2 (12.5%)	0 (0.0%)
Sleep disorder	2 (12.5%)	0 (0.0%)
Thyroid disorder	5 (31.3%)	3 (30.0%)
Resident county designation (%)^b^		
Micropolitan	7 (43.8%)	5 (50.0%)
Noncore isolated	9 (56.3%)	5 (50.0%)

**Notes:**

a Medical conditions were assessed by participant self-report.

b Resident county designation as defined by the NCHS Urban-Rural Classification Scheme for Counties 2013. Micropolitan has one urban cluster of at least 10,000 people but less than 50,000 people or 25% or more employed workforce commuting to this county. Noncore are nonmetropolitan counties that have no city, town or urban center with cluster of 10,000 residents or more.

c Too few males to report.

### Outcomes of objective 1: assessment and mitigation of barriers

#### Information technology access and literacy

Results of the information technology access and literacy survey are reported separately for total and non-completing participants (Table S5). All participants (*N* = 16) claimed access to a home computer and a smartphone. Fourteen of 16 (88%) of participants had home internet, but 31% (*N* = 5) preferred to use their workspace internet. Fifty-six percent of respondents claimed their internet connection was somewhat reliable (*N* = 2) or very unreliable (*N* = 7). The literacy portion enquiring on level of comfort, confidence, and ability to use information technology and devices resulted on average with most feeling “very comfortable,” “very confident,” and “very well.” There were no distinguishable differences between participants’ access to information technology or perceived technical literacy.

### Outcomes of objective 2: feasibility and acceptability

#### Feasibility outcomes

A summary of primary outcome measures is provided in Table 2. At 3 months, ten participants remained of which all completed the 12-month intervention, resulting in 62.5% retention. At 12-months, 62.5% of the eText sessions and 75% of the study measures were completed by participants (*N* = 16). Although not a primary outcome, for those participants that remained in the program (*N* = 10), completion of eText was 97.6% and of study measures was 100%.

**Table 2:** Primary outcome measures and results of feasibility and acceptability.

3-Month primary outcome measures	Results	
Achieve target enrollment of 16 participants	Enrolled 16 participants Recruitment duration 3 weeks	
Retention: Dropout <20%, 13 retained participants	Dropout 37.5%, 10 retained participants	
Acceptability: 4/5 on average for all program components^a^	Comprehensive program: 4.7 ± 0.3 MealPlot: 2.7 ± 0.9 eText: 4.4 ± 0.4 Nutrition coaching: 4.7 ± 0.4 Lifestyle coaching: 4.5 ± 0.7	
**12-Month primary outcome measures**	***N* = 16**	***N* = 10**
Completion of 75% eText	62.5%	97.6%
Completion of 80% of study measures^b^	75.0%	100.0%

**Notes:**

a Program components included were in regards to comprehensive program, MealPlot, eText, Nutrition Coaching collected *via* survey using Likert scales (ranging 1–5). Eleven of the 16 participants completed this survey at 3-months.

b Study measures including 24-h records, in-person anthropometric testing collected at baseline, 6- and 12-months. Numbers are presented for all participants (*N* = 16) and those that completed the study (*N* = 10).

#### Acceptability survey and interview

The acceptability survey and interview results are in Table 2. Survey answers were provided on a Likert-scale using ratings from 1–5 (1 = not satisfied/helpful, 5 = extremely satisfied/helpful) for each component of EMPOWER Rural program. The average satisfaction (*N* = 11) of the overall intervention was 4.7 ± 0.3, the MealPlot application was 2.7 ± 0.9, the eText was 4.4 ± 0.4, the nutrition coaching was 4.7 ± 0.4, and the lifestyle coaching was 4.5 ± 0.7. The interview portion of the acceptability measures included six open-ended questions enquiring on suggestions for improvement. There were several suggestions provided to improve the MealPlot web application. Eight of the eleven respondents said the search feature of the food database did not pull the results they needed to complete a food record. Ten respondents mentioned that the app was “cumbersome,” “difficult to use,” or “not intuitive.” The other components of the program (eText, nutrition coaching, lifestyle coaching) were provided with few comments for improvement which was consistent with their high Likert-scale satisfaction ratings. There was a high rate of voluntary participation in the optional lifestyle support components, which also supports adequate acceptability of the program. Among the ten participants who completed the program, participation was 100% in one or more lifestyle support components. Among all enrolled participants, 14/16 engaged in the lifestyle intake interview at baseline (86%), and 10/16 participants engaged in one or more advising sessions (63%), and also in one or more support group call-ins (63%). These rates surpass the benchmark of acceptability of at least 50% participation in one or more lifestyle components.

### Early indicators of efficacy

#### Anthropometric changes

On average, completing participants (*N* = 10) lost a clinically significant weight loss of 6.2 ± 6.0% (*p* = 0.014) body weight or 5.7 ± 5.3 kg (*p* = 0.014, d_RM_ = −1.78) from baseline to 12 months. Results can be seen in Table S6. Five participants achieved clinically significant weight loss of >5%, eight participants lost weight, one participant maintained the baseline weight, and one participant gained weight. Skeletal muscle was preserved with an average loss of 0.5 ± 1.2 kg. Most weight loss can be attributed to fat mass with an average loss of 4.9 ± 5.5 kg (*p* = 0.022, d_RM_ = −0.88). Individual changes in weight, body fat, and skeletal muscle mass can be found in Fig. S7.

#### Dietary changes

Diet changes for the Food Frequency Questionnaire (FFQ) and 24-h records from baseline to 12-months are in Table S8. For those who completed the Baseline Food Frequency Questionnaire (FFQ) and 12-months FFQ (*N* = 10), no significant changes to calorie, protein, or fiber intake were demonstrated; however, on average, there was a numerically lower amount of calories and higher amount of protein. The average calorie intake was numerically lower by 173.9 ± 584.9 kcal (*p* = 0.27, d_RM_ = −0.49). The average protein density was numerically higher by 0.94 ± 1.42 g/100 kcal (*p* = 0.06, d_RM_ = 1.01). There was a trend in rising fiber density on average although no significant change. The fiber density numerically was higher by 0.05 ± 0.34 g/100 kcal (*p* = 0.43, d_RM_ = 0.17).

A total of 24-h records at baseline and 12-months (*N* = 10) showed similar results to the FFQ with no significant changes to protein and fiber density; however, improving trends were observed, more notably to protein. Protein density increased from baseline to 12-months of 1.41 ± 2.63 g/100 kcal (*p* = 0.23, d_RM_ = 0.75).

#### Exit surveys

All remaining participants completed the Exit Survey (*N* = 10) providing feedback on the program (Table S9). Eight of the participants indicated nutrition coaching and lifestyle coaching to be their most favored components. Eight concluded that the EMPOWER program helped them to achieve or get closer to their weight goals. When inquired about suggestions for changing the program, answers were highly individual, however a common theme was increasing ease of use, such as shorter eText videos, more intuitive MealPlot web application, and fewer emails. All participants indicated that the program was accessible and that despite living in areas where cell and internet connection is unstable, the remote nature of the program was highly favorable. Five participants indicated that they would sustain the changes they made to either their diet or eating pattern, and two responded that they will continue weighing daily.

## Discussion

### Main findings

Of our five progression criteria, two were achieved by all participants as recruited and all five by those who completed the entire trial (Fig. S10). More participants dropped the program than anticipated and a clear juxtaposition was shown by the different amount of education sessions completed by those who remained in the program. Most participants claimed life stressors unrelated to the program prompted their exit. While our ability to achieve target enrollment was achieved, our other main hypothesis criterion for feasibility including 80% retention and 75% completion of education sessions and 80% study measures were not met due to a larger than anticipated dropout of 37.5%. The pilot study suggests EMPOWER may be an acceptable and efficacious weight loss program for rural residents based on outcomes of retained participants. Program components, including the nutrition and lifestyle education sessions were ranked highly acceptable and enjoyable. Like other rural weight loss interventions, the online platform improved accessibility and was well-liked (Batsis et al., 2019; Befort et al., 2021). Also, most participants who completed the program lost weight and improved their diet quality. Retained participants on average achieved a significant degree of weight loss of 6.2 ± 6.0% and maintained the loss throughout this long-term trial. Compared to other weight loss interventions in rural areas, we report a higher percentage of participants achieving medically significant weight loss, with 50% of participants achieving at least 5% loss at 12-months (Robinson et al., 2022; Batsis et al., 2019).

### Comparison with existing literature

Other studies also indicate that telehealth poses a promising method to remove barriers to healthcare for rural residents (Calancie et al., 2015; Chu et al., 2021). In a pilot study of a telehealth weight loss program for rural residents in New Hampshire, intervention participation, participant retention, and measures of acceptability met or surpassed *a priori* thresholds (Batsis et al., 2019). The program consisted of 16 weekly virtual sessions from either a dietitian, health coach, or exercise trainer modeled after the health-behavior change Diabetes Prevention Program (Batsis et al., 2019). Nineteen percent of completing participants lost on average 5% or more of their weight within the 16-week trial and retention was 75.7% (Batsis et al., 2019). The overall telehealth intervention was rated with high satisfaction of 4.48 ± 0.58 using a 5-point Likert scale (range 1–5, low-high satisfaction) (Batsis et al., 2019). Although rural areas have less internet and cell access, participants reported that remote delivery was more convenient and time saving (Batsis et al., 2019); However, lack of face-to-face contact was cited as a negative theme to telehealth (Batsis et al., 2019). Overall, this research indicates that telehealth presents a viable, well-received, and effective approach for promoting weight loss among rural inhabitants. However, it’s important to acknowledge certain constraints on its broader applicability, as the study’s participants were drawn from a medical facility that offers substantial healthcare access, a scenario not applicable to most rural dwellers. Also, this initial pilot project spanned 16 weeks, which may limit its capacity to reflect long-term outcomes.

### Strengths and limitations

Despite the rural setting, initial concerns of access to internet and information technology literacy were unfounded (Hampton et al., 2021; Abdon, William & Tandika, 2023; Graves et al., 2021). Although internet reliability was claimed mostly unreliable, participants found means to participate in the online program and remarked that the online nature was preferable. Additionally, there were no marked differences in technology access or literacy between participants who remained and dropped the program and therefore, we believe this not a barrier for rural resident participation. However, there are limitations in the generalizability of this study especially concerning older populations who were underrepresented in our sample and have been cited in some literature to have less success with online intervention (Heponiemi et al., 2022; Kim, Oh & Shin, 2020).

The acceptability survey and exit interview provided valuable insight as to how the program may be improved, especially in regard to the web application, MealPlot which was ranked the least favorable component. The rural pilot was the first EMPOWER cohort required to use the MealPlot web application for reporting food records which proved to be a challenging feature for most participants. Other studies in rural areas report positive feasibility, acceptability, and weight loss using mobile and web applications suggesting that use of a web application such as MealPlot is plausible in this population, but that our app requires improvement (Batsis et al., 2020; Eisenhauer et al., 2021). Additionally, the small sample size of the exit interview must be acknowledged. We lack insight from the 22 participants who declined participation as to their reason for the decline. Comparably, other rural areas report far fewer declining participants (Batsis et al., 2020; Eisenhauer et al., 2021). Although we can only hypothesize, we believe that the consent form may have been overwhelming in technical jargon and length (Kurtz-Rossi et al., 2024).

Numerous weight loss trials traditionally draw participants from medical institutions or academic settings. However, our recruitment strategy *via* Extension was essential in guaranteeing the inclusion of participants from rural areas, which might lack access to such conventional services. The samples’ reported income, education, and weight-related comorbidities of the recruited population reflected expectations based on Illinois Behavioral Risk Factor Surveillance System data of 2015–2019 (Illinois Department of Public Health (IDPH), 2019). However, our sample included only one male and most participants were of middle age, which may have biased our results (Woźniak et al., 2022). Additionally, using Extension educators as recruiters may not be feasible for larger scale trials in the future as it is an added task to their already busy jobs.

## Conclusions

This study has provided crucial insights on how to provide rural communities with a weight loss program option that is feasible and acceptable. As it stands, there is limited justification for proceeding to a full-scale trial. However, exploratory analysis indicates that refining and retesting this intervention in a future feasibility trial may be worthwhile. Such a trial for rural populations would sustain the pilot’s features of online access, recruitment by Extension educators, and coaching by qualified professionals in nutrition and lifestyle as these were key factors in the pilot study’s success. Potential methods of improving retention have been identified, such as decreasing stressors of the program on rural residents by increasing usability of the web application and sending materials such as scales to participants’ homes instead of requiring them to travel to their local Extension office. In future research, a perpetual means of collaboration and recruitment will be necessary to expand in a way that does not burden Extension educators as recruiters and engages a more diverse group of ages and genders.

## Supplemental Information

10.7717/peerj.18268/supp-1 Supplemental Information 1 Protein and Fiber Chart. Protein Fiber Plot (PF Plot) with examples of foods plotted. Individual foods are plotted with colors indicating their protein and/or fiber density. An individual food that is high in protein and/or fiber density is green, moderate in protein and/or fiber density is yellow, and low in protein and/or fiber density is red. The green box represents the target area for the total meal which is represented as a purple dot. Dot size indicates the number of calories consumed within the plotted meal. Meals with mostly green foods will have a total that falls into the target, while red foods will draw the total away from the target.

10.7717/peerj.18268/supp-2 Supplemental Information 2 Weight Chart. Weight chart example as portrayed in the MealPlot web application. Individual users connect their Wi-Fi enabled scale to the web application to automatically load weights to the chart. The blue line represents the actual weights. The red line is a target trendline for the recommended 1 lb. per week weight loss. The user’s goal weight is entered into the chart by their nutrition coach, represented by the orange line. The goal weight is based on 1 lb. of loss per week over 3 months’ time, or 12.5 lbs. (5.7 kg). Once a user is close to their healthy weight, a nutrition coach will add the health weight target, represented by a green line.

10.7717/peerj.18268/supp-3 Supplemental Information 3 Questions provided at the 3 Months Acceptability Survey and Interview.

10.7717/peerj.18268/supp-4 Supplemental Information 4 Future Directions EMPOWER in Rural Areas Participant Exit Survey. Questions inquired at the 1-year Exit Survey

10.7717/peerj.18268/supp-5 Supplemental Information 5 Information Technology Access and Literacy Survey Results. Results of the Information Technology Access and Literacy Survey provided to participants at baseline. Completing participants are displayed by those participants who dropped the program.

10.7717/peerj.18268/supp-6 Supplemental Information 6 Anthropometric Measurements at Baseline and 12-Months. BMI: body mass index, M: mean, SD: standard deviation, Effect size: Cohen’s d for repeated measures, CI: confidence interval, p-value 0.05

10.7717/peerj.18268/supp-7 Supplemental Information 7 Individual weight, fat mass, and skeletal muscle mass changes from baseline to 12-months. Individual participant changes in weight, fat mass, and skeletal muscle mass as taken by bioimpedance.

10.7717/peerj.18268/supp-8 Supplemental Information 8 Diet changes from baseline to 12-Months. Diet changes from baseline to 12-months collected by Food Frequency Questionnaire (FFQ) and 24-hour record. M: mean, SD: standard deviation, Effect size: Cohen’s d for repeated measures, CI: confidence interval, p-value 0.05

10.7717/peerj.18268/supp-9 Supplemental Information 9 Exit survey feedback on the EMPOWER program from participants. Substantive quotes and themes from the exit survey (N=10) as collected and analyzed in evaluation of the EMPOWER program’s feasibility.

10.7717/peerj.18268/supp-10 Supplemental Information 10 Completed online nutrition education sessions per participant. Online nutrition education (eText) sessions completed by each participant by the end of the 1-year study. The red box indicates the participants who dropped the program (n=6).

10.7717/peerj.18268/supp-11 Supplemental Information 11 24 Hour Record Raw Data. Raw data from 24-hour food collection at baseline, 3 months, 6 months, and 1 year.

10.7717/peerj.18268/supp-12 Supplemental Information 12 Food Frequency Questionnaire raw data. Food Frequency Questionnaire raw data for the participants that remained the duration of the 1 year trial. Baseline and 1-year data is provided.

10.7717/peerj.18268/supp-13 Supplemental Information 13 Body composition and anthropometric raw data. Body composition, weight, waist and hip circumference, measurements of participants at baseline, 6 months, and 1 year.

10.7717/peerj.18268/supp-14 Supplemental Information 14 CONSORT extension Pilot and Feasibility Trials Checklist.

10.7717/peerj.18268/supp-15 Supplemental Information 15 Consort Flow Diagram.

## Additional Information and Declarations

### Data Availability

The following information was supplied regarding data availability: The weight measurements and diet record analysis at time points of data collection over the one-year study are available in the Supplemental Files.

### Clinical Trial Registration

The following information was supplied regarding Clinical Trial registration: NCT05587790
