# Supplementary figures and images for: Feasibility and acceptability pilot study of an online weight loss program in rural, underserved communities
Source: PeerJ. 2024 Oct 3;12:e18268. doi: 10.7717/peerj.18268 (PMC11456290; doi:10.7717/peerj.18268)

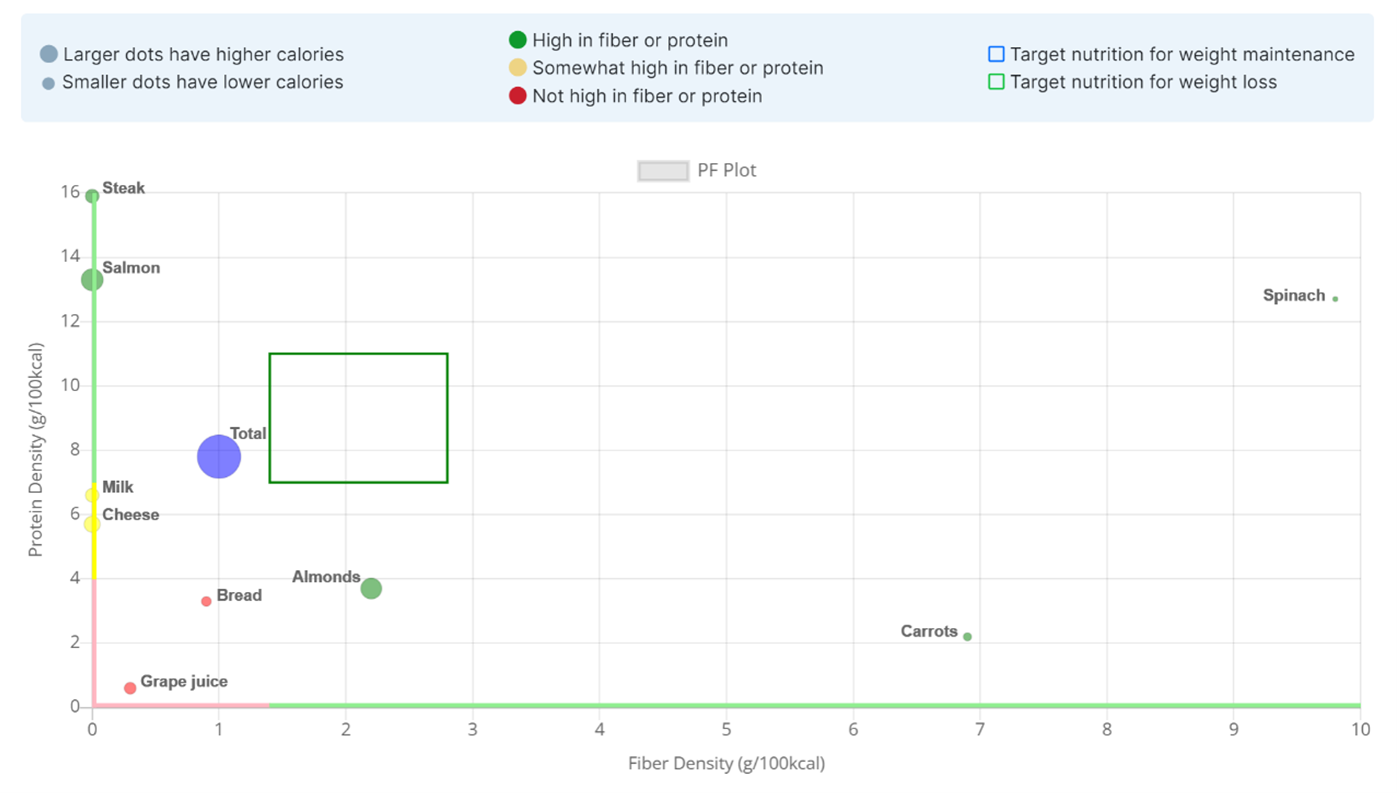

Supplement: Supplemental Information 1 — Protein Fiber Plot (PF Plot) with examples of foods plotted. Individual foods are plotted with colors indicating their protein and/or fiber density. An individual food that is high in protein and/or fiber density is green, moderate in protein and/or fiber density is yellow, and low in protein and/or fiber density is red. The green box represents the target area for the total meal which is represented as a purple dot. Dot size indicates the number of calories consumed within the plotted meal. Meals with mostly green foods will have a total that falls into the target, while red foods will draw the total away from the target. [file peerj-12-18268-s001.png]

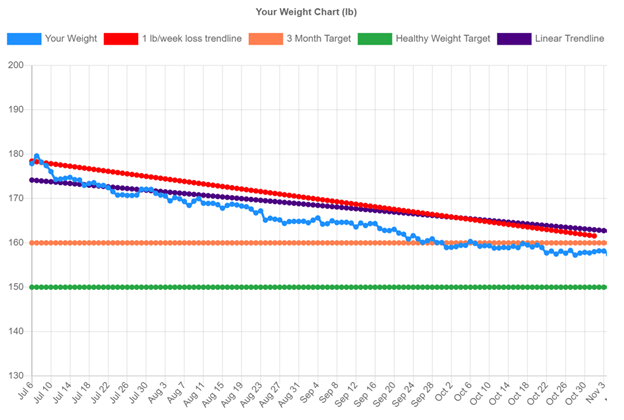

Supplement: Supplemental Information 2 — Weight chart example as portrayed in the MealPlot web application. Individual users connect their Wi-Fi enabled scale to the web application to automatically load weights to the chart. The blue line represents the actual weights. The red line is a target trendline for the recommended 1 lb. per week weight loss. The user’s goal weight is entered into the chart by their nutrition coach, represented by the orange line. The goal weight is based on 1 lb. of loss per week over 3 months’ time, or 12.5 lbs. (5.7 kg). Once a user is close to their healthy weight, a nutrition coach will add the health weight target, represented by a green line. [file peerj-12-18268-s002.png]

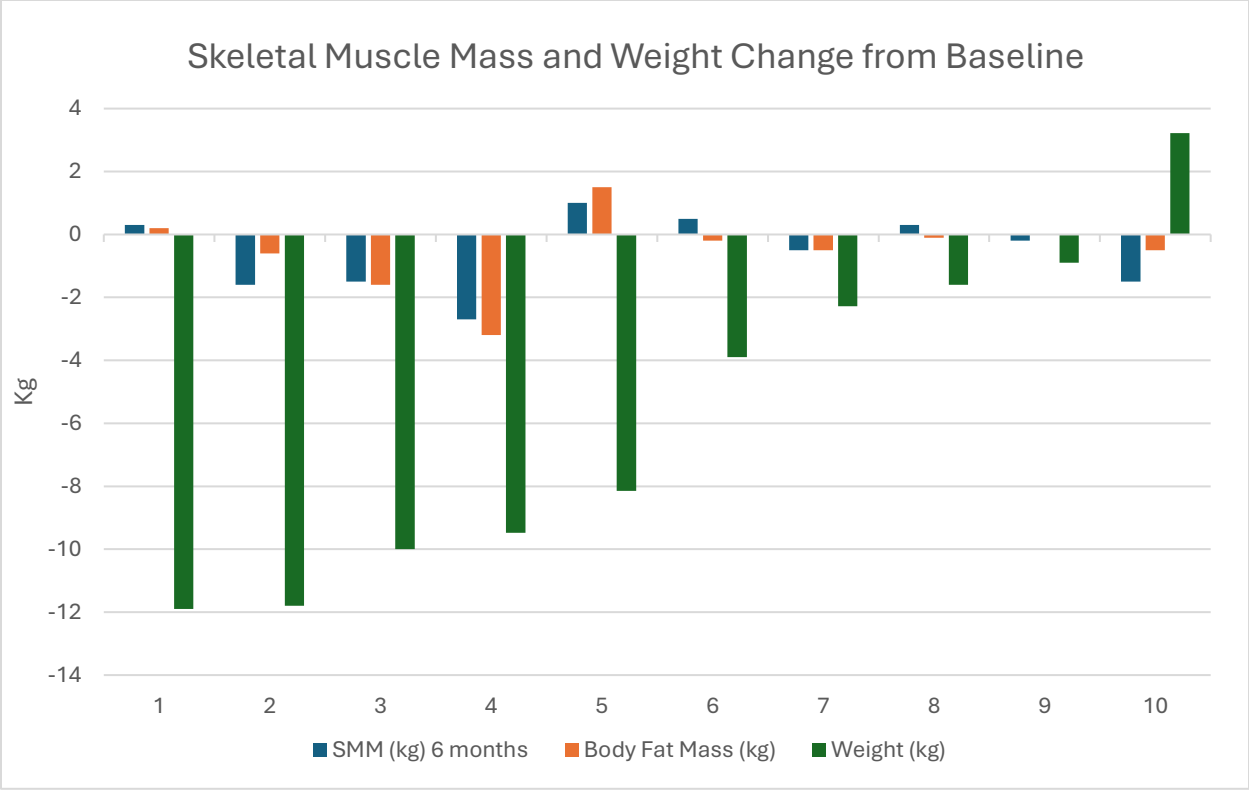

Supplement: Supplemental Information 7 — Individual participant changes in weight, fat mass, and skeletal muscle mass as taken by bioimpedance. [file peerj-12-18268-s007.pdf]

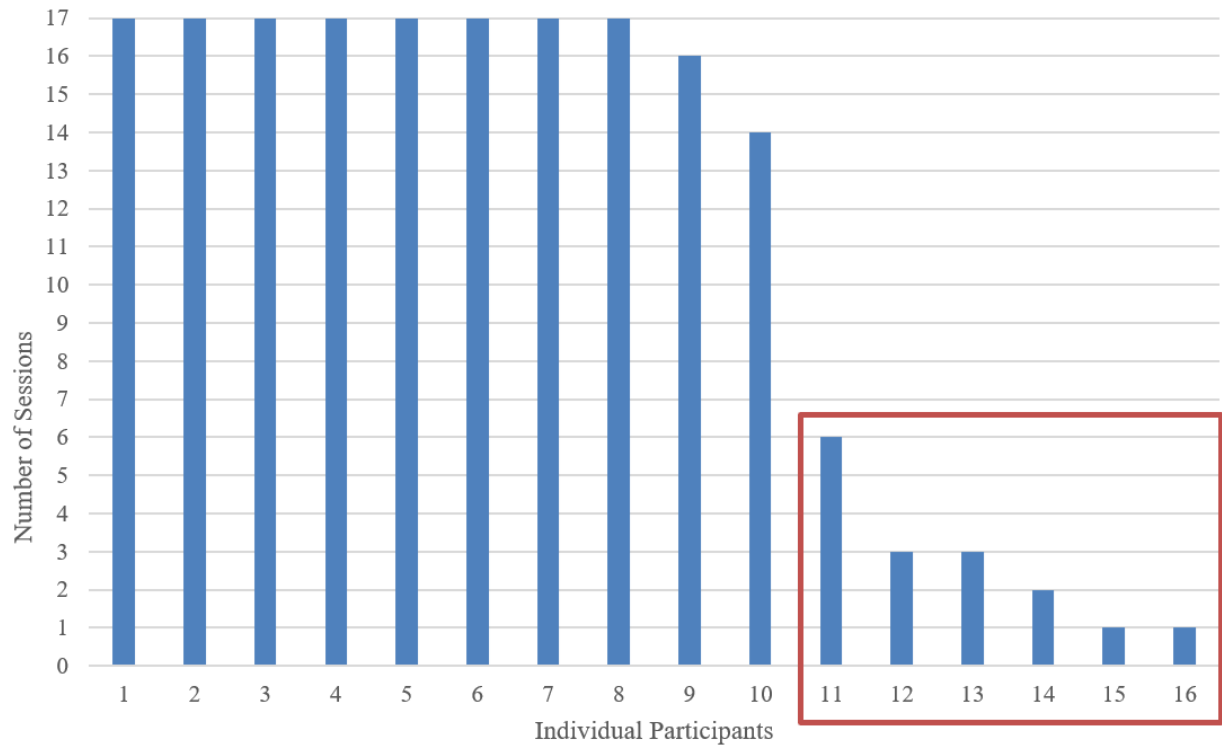

Supplement: Supplemental Information 10 — Online nutrition education (eText) sessions completed by each participant by the end of the 1-year study. The red box indicates the participants who dropped the program (n=6). [file peerj-12-18268-s010.pdf]

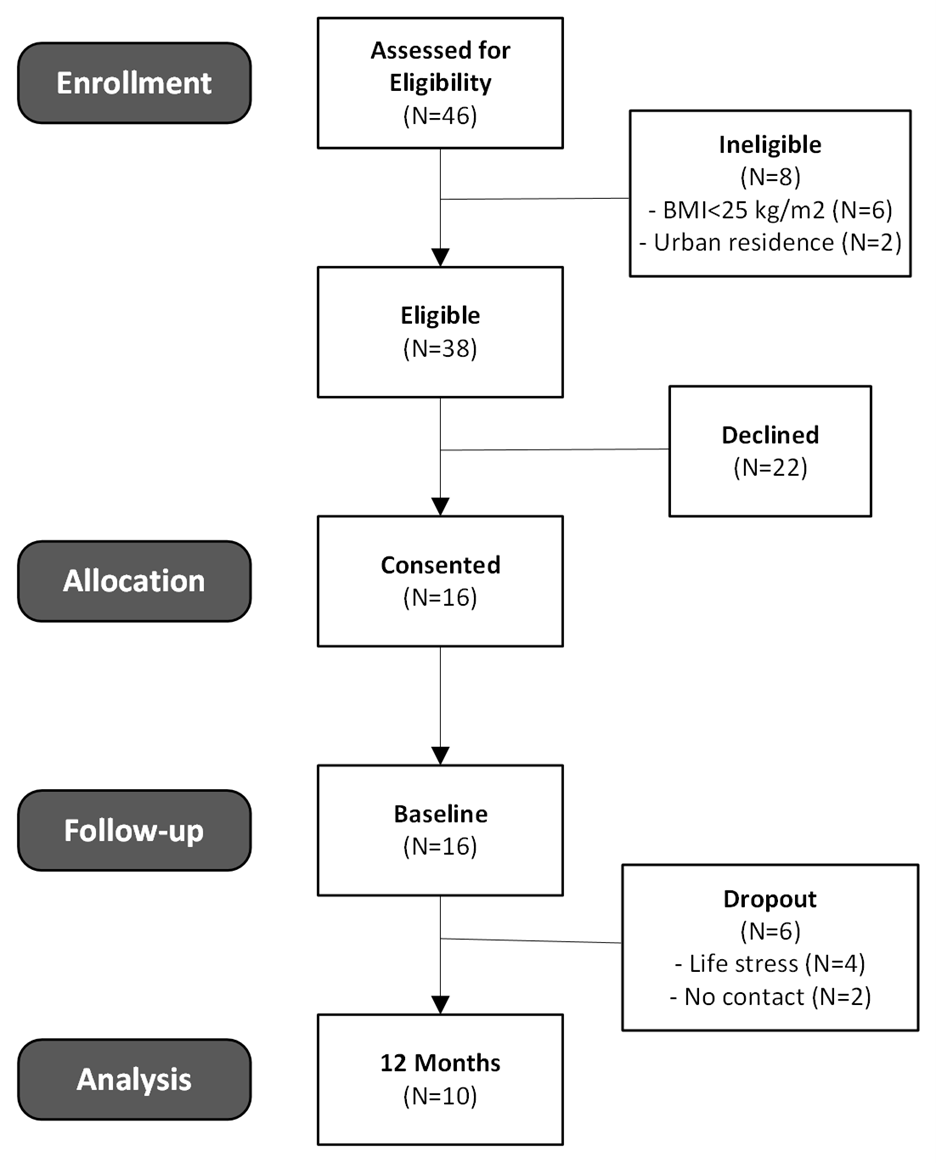

Supplement: Supplemental Information 15 [file peerj-12-18268-s015.docx]
