# Supplementary material for: Feasibility and acceptability pilot study of an online weight loss program in rural, underserved communities
Source: PeerJ. 2024 Oct 3;12:e18268. doi: 10.7717/peerj.18268 (PMC11456290; doi:10.7717/peerj.18268)
Supplement: Supplemental Information 3 [file peerj-12-18268-s003.pdf]

3-MONTHS ACCEPTABILITY SURVEY AND INTERVIEW

Overall Intervention

1. How would you rate your level of **satisfaction** with the overall intervention itself? (5=extremely satisfied, 1=not satisfied)
2. Would you **recommend** this intervention to a family member or friend who has a weight problem? (5=highly recommend, 1=would not recommend)
3. Do you feel that a virtual (online) weight loss program is **helpful** for patients that live in rural areas? (5=extremely helpful, 1=not helpful)
4. Has the program been **helpful** in achieving your goal? (5=extremely helpful, 1=not helpful)

MealPlot (www.MealPlot.com, One Day Record, Weight chart, Meal Planner)

1. How would you rate your level of **satisfaction** with MealPlot? (5=extremely satisfied, 1=not satisfied)
2. How **helpful** is MealPlot in assisting you to achieve your goals? (5=extremely helpful, 1=not helpful)
3. Do you find MealPlot **easy to use** without much difficulty? (yes, no)
4. Do you feel that you had adequate support to use and/or ask questions in relation to this MealPlot? (yes, no)
5. How can we improve MealPlot to make your experience better? ______

eText (online education videos and readings)

1. How would you rate your level of **satisfaction** with the eText? (5=extremely satisfied, 1=not satisfied)
2. How would you rate your level of **satisfaction** with the length of the eText? (5=extremely satisfied, 1=not satisfied)
3. How would you rate your level of **satisfaction** with the activities in the eText (Make a Change)? (5=extremely satisfied, 1=not satisfied)
4. How **helpful** is the eText in assisting you to achieve your goals? (5=extremely helpful, 1=not helpful)
5. How can we improve the eText to make your experience better? ______

Nutrition Coaching

1. How would you rate your level of **satisfaction** with the nutrition coaching? (5=extremely satisfied, 1=not satisfied)
2. How would you rate your level of **satisfaction** with the amount of interaction with a nutrition coach? (5=extremely satisfied, 1=not satisfied)
3. How **helpful** is the nutrition coaching in assisting you to achieve your goals? (5=extremely helpful, 1=not helpful)
4. How can we improve the nutrition coaching to make your experience better? ______

Lifestyle/Social Work Coaching

1. How would you rate your level of **satisfaction** with the lifestyle coaching? (5=extremely satisfied, 1=not satisfied)
2. How would you rate your level of **satisfaction** with the amount of interaction with a social work lifestyle coach? (5=extremely satisfied, 1=not satisfied)

3. How **helpful** is the lifestyle coaching in assisting you to achieve your goals? (5=extremely helpful, 1=not helpful)
4. How can we improve the lifestyle coaching to make your experience better? ______
