# Supplementary material for: Feasibility and acceptability pilot study of an online weight loss program in rural, underserved communities
Source: PeerJ. 2024 Oct 3;12:e18268. doi: 10.7717/peerj.18268 (PMC11456290; doi:10.7717/peerj.18268)
Supplement: Supplemental Information 4 — Questions inquired at the 1-year Exit Survey [file peerj-12-18268-s004.pdf]

Future Directions EMPOWER in Rural Areas Participant Exit Survey

"Thank you for your participation in our EMPOWER weight loss program! I would like to take 10 to 15 minutes of your time to get your feedback on the program. I will not be recording your name, nor any personal identifiers and this interview will in no way be traceable to you besides my own recollection."

EMPOWER Program Feedback

1. Of the following parts of our program, which did you enjoy the most (MealPlot, nutrition coaching, lifestyle coaching, online education videos and text)?
2. Of the following parts of our program, which did you least enjoy (MealPlot, nutrition coaching, lifestyle coaching, community call-in support group, online education videos and text)?
3. Did the EMPOWER program help you to achieve or get closer to your weight goals?
4. If you could change the program in any way, what would you change?
5. Did you find the program accessible? (probes: time commitment, cost, energy, ease of use, ease of understanding, internet, modes of communication)
6. What changes, if any, have you made because of the program that you will continue?

"Thank you for taking the time to talk with me. Your feedback and participation in this program have helped us to advance our research and is truly invaluable."
