# Supplementary material for: Feasibility and acceptability pilot study of an online weight loss program in rural, underserved communities
Source: PeerJ. 2024 Oct 3;12:e18268. doi: 10.7717/peerj.18268 (PMC11456290; doi:10.7717/peerj.18268)
Supplement: Supplemental Information 5 — Results of the Information Technology Access and Literacy Survey provided to participants at baseline. Completing participants are displayed by those participants who dropped the program. [file peerj-12-18268-s005.pdf]

Information Technology Access and Literacy Survey Results

Question	Answer	Completing Participants (N=10)	Dropped participants (N=6)
**<u>Device and Internet Access</u>**			
**Home computer**	Yes	10, 100%	6, 100%
**Tablet**	Yes	6, 60%	2, 33%
	No	4, 40%	4, 67%
**Smartphone**	Yes	10, 100%	6, 100%
	No	0, 0%	0, 0%
**Home internet**	Yes	8, 80%	6, 100%
	No	2, 20%	0, 0%
**Public internet**	Yes	4, 40%	3, 50%
	No	6, 60%	3, 50%
**Preferred space for using internet**	Home	3, 30%	6, 100%
	Work	5, 50%	0, 0%
**Reliability of internet**	Very reliable	1, 10%	2, 33%
	Reliable	2, 20%	0, 0%
	Somewhat reliable	1, 10%	1, 17%
	Very unreliable	4, 40%	3, 50%
**<u>Information technology literacy</u>**			
**Comfort using a computer**	Very comfortable	7, 70%	6, 100%
	Comfortable	2, 20%	0, 0%
	Very uncomfortable	1, 10%	0, 0%
**Comfort using a smartphone**	Very comfortable	7, 70%	5, 83%
	Comfortable	2, 20%	1, 17%
	Very uncomfortable	1, 10%	0, 0%
**Comfort using the internet**	Very comfortable	7, 70%	6, 100%
	Comfortable	2, 20%	0, 0%
	Very uncomfortable	1, 10%	0, 0%
**Overall comfort using**	Very comfortable	5, 50%	4, 67%

**information technology**	Comfortable	5, 50%	2, 33%
**Confidence using Zoom**	Very confident	6, 60%	3, 50%
	Confident	3, 30%	2, 33%
	Very unconfident	1, 10%	1, 17%
**Confidence smartphone applications**	Very confident	8, 80%	6, 100%
	Confident	2, 20%	0, 0%
**Overall confidence using telehealth, websites, and smartphone applications**	Very confident	6, 60%	6, 100%
	Confident	3, 30%	0, 0%
	Somewhat confident	1, 10%	0, 0%
**Ability to open and send**	Very well	10, 100%	6, 100%
**Ability to forward an email**	Very well	10, 100%	6, 100%
**Ability to open an email**	Very well	10, 100%	6, 100%
**Ability to use a search engine**	Very well	8, 80%	6, 100%
	Well	2, 20%	0, 0%
**Ability to bookmark a webpage**	Very well	8, 80%	6, 100%
	Well	2, 20%	0, 0%
**Ability to move back and forth between web pages**	Very well	7, 70%	4, 67%
	Well	2, 20%	2, 33%
	Okay	1, 10%	0, 0%
**Ability to save a document**	Very well	10, 100%	6, 100%
**Overall ability to use information technology**	Very well	8, 80%	6, 100%
	Well	2, 20%	0, 0%
