# Supplementary material for: Feasibility and acceptability pilot study of an online weight loss program in rural, underserved communities
Source: PeerJ. 2024 Oct 3;12:e18268. doi: 10.7717/peerj.18268 (PMC11456290; doi:10.7717/peerj.18268)
Supplement: Supplemental Information 6 — BMI: body mass index, M: mean, SD: standard deviation, Effect size: Cohen’s d for repeated measures, CI: confidence interval, p-value 0.05 [file peerj-12-18268-s006.pdf]

	Baseline (N=10)	12-months (N=10)	Change	P-value	Effect Size (95% CI)
	M ± SD	M ± SD	M ± SD		
Weight, kg	99.9 ± 14.6	94.2 ± 18.1	-5.7 ± 5.3	0.0137	-1.78 (-2.81, -0.74)
Fat mass, kg	46.2 ± 9.7	41.4 ± 13.4	-4.9 ± 5.5	0.0215	-0.88 (-1.80, 0.04)
Skeletal muscle mass, kg	30.0 ± 5.2	29.5 ± 4.5	-0.5 ± 1.2	0.1406	-0.62 (-1.52, 0.28)
BMI, kg/m2	37.5 ± 4.4	35.8 ± 4.4	-1.7 ± 1.8	0.0039	-0.69 (-1.59, 0.21)
Waist circumference, cm	113.8 ± 9.2	108.2 ± 12.4	-5.6 ± 6.2	0.0391	-1.18 (-2.13, -0.23)
Hip circumference, cm	120.8 ± 8.4	115.8 ± 13.2	-5.0 ± 4.2	0.1094	-0.74 (-1.64, 0.17)
