# Supplementary material for: Feasibility and acceptability pilot study of an online weight loss program in rural, underserved communities
Source: PeerJ. 2024 Oct 3;12:e18268. doi: 10.7717/peerj.18268 (PMC11456290; doi:10.7717/peerj.18268)
Supplement: Supplemental Information 8 — Diet changes from baseline to 12-months collected by Food Frequency Questionnaire (FFQ) and 24-hour record. M: mean, SD: standard deviation, Effect size: Cohen’s d for repeated measures, CI: confidence interval, p-value 0.05 [file peerj-12-18268-s008.pdf]

	Baseline (N=10)	12-months (N=10)	Change	P-value	Effect Size (95% CI)
	M ± SD	M ± SD	M ± SD		
Food Frequency Questionnaire (FFQ)					
Calories, kcal	2033.4 ± 409.1	1859.5 ± 675.2	-173.9 ±584.9	0.2754	-0.49 (-1.38, 0.40)
Fiber, g	19.4 ± 6.5	18.1 ± 6.4	-1.4 ± 4.1	0.5566	-0.41 (-1.29, 0.48)
Protein, g	101.3 ± 19.0	114.4 ± 61.3	13.1 ± 57.8	0.7695	0.68 (-0.23, 1.58)
Fiber density, g/100kcal	0.96 ± 0.26	1.01 ± 0.29	0.05 ± 0.34	0.4316	0.17 (-0.71, 1.05)
Protein density, g/100kcal	5.04 ± 0.73	5.98 ± 1.30	0.94 ± 1.42	0.0645	1.01 (0.08, 1.94)
24-hour Record					
Calories, kcal	1862.8 ± 426.2	1374.4 ± 367.9	-488.4 ± 567.6	0.0195	-0.87 (-1.79, 0.04)
Fiber, g	21.2 ± 6.2	17.3 ± 8.0	-3.9 ± 8.9	0.2500	-0.46 (-1.35, 0.43)
Protein, g	86.0 ± 20.9	82.9 ± 28.4	-3.1 ± 35.9	0.4316	-0.10 (-0.98, 0.77)
Fiber density, g/100kcal	1.19 ± 0.41	1.21 ± 0.47	0.02 ± 0.59	0.7520	0.03 (-0.84, 0.91)
Protein density, g/100kcal	4.9 ± 1.6	6.3 ± 2.3	1.41 ± 2.63	0.2324	0.75 (-0.16, 1.66)
