# Supplementary material for: Feasibility and acceptability pilot study of an online weight loss program in rural, underserved communities
Source: PeerJ. 2024 Oct 3;12:e18268. doi: 10.7717/peerj.18268 (PMC11456290; doi:10.7717/peerj.18268)
Supplement: Supplemental Information 9 — Substantive quotes and themes from the exit survey (N=10) as collected and analyzed in evaluation of the EMPOWER program’s feasibility. [file peerj-12-18268-s009.pdf]

Q: Of the following parts of our program, which did you enjoy the most (MealPlot, nutrition coaching, lifestyle coaching, online education videos and text)?

8/10 participants' favorite indicated as nutrition, lifestyle, or general coaching.

"Nutrition coaching…(the coach's) nutrition plan and the accountability of seeing someone for me works really well."

"Lifestyle coaching. I enjoyed the nutrition coaching too, but I really connected with (lifestyle coaches)."

Q: Of the following parts of our program, which did you least enjoy (MealPlot, nutrition coaching, lifestyle coaching, online education videos and text)?

10/10 participants indicated MealPlot or a feature of MealPlot.

"MealPlot. Some of the features. I used the weight chart. I didn't get the one-day record page at all."

"MealPlot. You should consider older adults. This is too hard for us."

Q: Did the EMPOWER Rural program help you to achieve or get closer to your weight goals?

8/10 participants responded yes.

"Yes, definitely. It took me a while to get into this program for some reason and so I'm not at my goal yet, but now I'm doing really well. I want to keep going."

"I think so. I think I still have a way to go, but I'm proud of where I'm at now."

Q: If you could change the program in any way, what would you change?

8/10 participants indicated a need for increased ease of use in one or more program components.

"Maybe simplify it. I have so much going on and it was hard to keep track of all the stuff flooding my emails. I started just ignoring them at times."

"Maybe you could make videos shorter or make it audible."

Q: Did you find the program accessible? (probes: time commitment, cost, energy, ease of use, ease of understanding, internet, modes of communication)

10/10 participants responded yes.

"Yeah. Our internet is terrible, but I would go to the library and bang out a bunch of sessions and emails."
"Yes, I like that I don't have to drive anywhere or buy anything."

Q: What changes, if any, have you made because of the program that you will continue?

5/10 participants indicated diet or eating patterns.

"I have a routine now which I've never had in my whole life. I plan my foods and I eat at the same times."

"The eating window is a biggy. I also don't snack as frequently."
